# Patients’ and Health Care Professionals’ Perceptions of the Potential of Using the Digital Diabetes Questionnaire to Prepare for Diabetes Care Meetings: Qualitative Focus Group Interview Study

**DOI:** 10.2196/17504

**Published:** 2020-08-19

**Authors:** Katarina Eeg-Olofsson, Unn-Britt Johansson, Ebba Linder, Janeth Leksell

**Affiliations:** 1 Sahlgrenska University Hospital Gothenburg Sweden; 2 Institute of Medicine Department of Molecular and Clinical Medicine University of Gothenburg Gothenburg Sweden; 3 Sophiahemmet University Stockholm Sweden; 4 Department of Clinical Sciences and Education Karolinska Institutet Stockholm Sweden; 5 Center of Registers Västra Götaland Nationella Diabetesregistret Gothenburg Sweden; 6 School of Education, Health and Social Studies Dalarna University Falun Sweden; 7 Department of Medical Sciences, Clinical Diabetology and Metabolism Uppsala University Uppsala Sweden

**Keywords:** Digital questionnaire, health care professionals, diabetes care, focus group interview, qualitative research, eHealth

## Abstract

**Background:**

In effective diabetes management, it is important that providers and health care systems prioritize the delivery of patient-centered care and that they are respectful of and responsive to individual patient preferences and barriers.

**Objective:**

The objective of the study was to conduct focus group interviews to capture patients’ and health care professionals’ perceptions and attitudes regarding digital technology and to explore how the digital Diabetes Questionnaire can be used to support patient participation in diabetes care, as a basis for an implementation study.

**Methods:**

A qualitative study was conducted with six focus group discussions with diabetes specialist nurses and medical doctors (n=29) and four focus group discussions with individuals with diabetes (n=23). A semistructured focus group interview guide was developed, including probing questions. The data were transcribed verbatim, and qualitative content analysis was performed using an inductive approach.

**Results:**

Two main categories were revealed by the qualitative analysis: *perceptions of digital technology and the digital questionnaire in diabetes management and care* and *perceptions of participation in diabetes care*. An overarching theme that emerged from the focus group interviews was *patients’ and professionals’ involvement in diabetes care using digital tools.*

**Conclusions:**

The analysis identified important factors to consider when introducing the digital Diabetes Questionnaire in clinical use. Both professionals and patients need support and training in the practical implementation of the digital questionnaire, as well as the opportunity to provide feedback on the questionnaire answers.

## Introduction

### Background

In effective diabetes management, it is important that providers and health care systems prioritize the delivery of patient-centered care, acknowledging multiple morbidity and being respectful of and responsive to individual patients’ preferences and barriers, including the differential costs of therapies [1]. Practicing patient-centered psychosocial care requires that the context of the person with diabetes be considered in communications and interactions, problem identification, psychosocial screening, diagnostic evaluation, and intervention services [2].

### Importance of Patient-Reported Outcomes

In line with these requirements for patient-centered care, our research group has developed and psychometrically tested a digital patient-reported outcome measure, ie, the Diabetes Questionnaire [3-7]. Patients respond digitally to the Diabetes Questionnaire, and these responses form the basis for the patient’s and the health care staff’s preparation to be well-informed to discuss the patient’s care and make decisions based on the patient’s wishes, needs, and barriers. The purpose of the Diabetes Questionnaire is to be user-friendly and immediately provide results to the patients and health care professionals as part of the consultation. The Diabetes Questionnaire is a tool for person-centered care that creates the necessary conditions for shared, well-founded decisions regarding patients living with diabetes. A recent review by Skovlund et al [8] concluded that patient-reported outcome measures have the potential to facilitate person-centered care and active participation. The long-term goal is for the Diabetes Questionnaire to be used to capture patient-reported outcome measures in integrated diabetes care and contribute to better health care meetings, facilitate thorough follow-up over time, and be considered together with the medical variables in the National Quality Registry for Diabetes.

### Challenges and Possibilities for Digital Health Tools

Digital developments in health care have created a great need for research on how digital health care services affect, for example, health care quality, design, and accessibility. Despite great interest from users, decision makers, and academics, the research base on these issues is limited [9]. The research available in this area focuses mainly on specific implementations, such as the psychotherapeutic treatment of mental illnesses or the diagnosis of skin diseases using digital tools, in more traditional telemedicine or in related areas such as online journals [10]. However, more research is needed on digital health care services, both because many conditions that are currently handled in digital health care services have not yet been investigated and because new digital tools that can change digital and physical health care services and care processes are constantly being developed and need to be evaluated separately [11,12]. In this study, we defined digital health as “the cultural transformation of how disruptive technologies that provide digital and objective data accessible to both caregivers and patients leads to an equal level doctor-patient relationship with shared decision-making and the democratization of care [13].”

### Rationale

The rationale of this study was based on earlier studies and reviews [14,15] that pointed out that the basis of knowledge for conducting an implementation study of patient-reported outcome measures should be tailored by identifying and addressing potential barriers and facilitators specific to the setting. In addition, the methodological quality of existing evidence with respect to digital health interventions for chronic diseases such as diabetes is low, and the results are unpredictable [16]. As a first step, we therefore decided to conduct focus group discussions with both patients and professionals involved in diabetes care, to inform a forthcoming implementation study of the Diabetes Questionnaire in a diabetes care setting.

### Aim of the Study

The purpose of this study was to conduct focus group interviews to capture patients’ and health care professionals’ perceptions and attitudes regarding digital technology and to explore how the digital Diabetes Questionnaire can be used to support patient participation in diabetes care, as the basis for an implementation study.

## Methods

### Research Design

An exploratory and descriptive qualitative design was used in this study. The data were collected through focus group discussions conducted from June 2018 to November 2018 with diabetes specialist nurses and medical doctors and with adults with type 1 diabetes or type 2 diabetes.

Focus groups have been widely used to examine persons’ experiences, and this method was chosen because the focus group environment is socially oriented and may increase the sense of belonging and cohesiveness among the participants, which can lead to increased openness [17].

### Sample and Setting

The participants were recruited through purposive sampling, which involves a conscious selection of individuals with the appropriate experiences or characteristics [18]; 18 hospital-based outpatient clinics and 22 primary health care clinics were initially approached. Of these, 14 hospital-based outpatient clinics and 8 primary health care clinics agreed to participate in the study.

The study was approved by the Regional Ethical Review Board in Gothenburg, Sweden (No. 317-18). A letter to the participants informed them about the study’s purpose, the voluntary nature of their participation, the confidentiality measures and methods of handling of their personal data, the National Diabetes Register, contact details, and the right to end participation. All participants gave written informed consent, and the research was performed in accordance with the Declaration of Helsinki [19].

### Participants and Procedure

The focus group discussions were held at hospital outpatient centers, primary health care clinics, and the Center of Registers Västra Götaland. Each group consisted of 3 to 6 participants; 6 focus group discussions were held with diabetes specialist nurses and medical doctors (health care professionals), and 4 focus group discussions were conducted with persons with type 1 diabetes or type 2 diabetes (patients). Focus group participant characteristics are presented in Table 1.

At the beginning of each focus group discussion, the participants completed a brief questionnaire that asked about their demographic characteristics (age, gender, education, occupation, age at diagnosis, and type of diabetes). The discussion opened with a general introduction of the study, an overview of the purpose of the discussion, and its confidentiality. The duration of the focus group discussions ranged from 0.6 hours to 1.5 hours. KEO or JL moderated the focus groups, and EL facilitated the discussions. All participants were given sufficient opportunity to share their views. We conducted separate focus group discussions for patients and for health care professionals. A semistructured focus group interview guide was developed according to the study aims (Multimedia Appendix 1). Furthermore, probing questions were used, for example, “Could you please further describe the situation using a concrete example?” All focus group discussions were audio-recorded with a digital voice recorder and transcribed verbatim by a medical secretary.

**Table 1:** Focus group participant characteristics.

Participant characteristics			Value
**Patients with diabetes (4 focus groups, n=23)**			
	Age (years), median (range^a^)		60 (22-81)
	**Gender, n (%)**		
		Women	11 (48)
		Men	12 (52)
	Diabetes duration (years), median (range)		21 (3-64)
	**Diabetes type, n (%)**		
		Type 1	17 (74)
		Type 2	6 (26)
**Health-care professionals (6 focus groups, n=29)**			
	Age (years), median (range)		53 (36-70)
	**Gender, n (%)**		
		Women	24 (83)
		Men	5 (17)
	**Role, n (%)**		
		Diabetes specialist nurses	23 (79)
		Medical doctors	6 (21)

^a^Minimum to maximum.

### Data Analysis

Qualitative content analysis inspired by Krippendorff [20] was used with an inductive approach. The focus group discussions and content analysis were performed in Swedish. The research group has deep knowledge and experience in this method. The quotations presented in this paper were translated into English by a professional translator. The data analysis was manually performed as follows:

Step 1: The transcribed focus groups discussions were read through several times to obtain an overall sense of the data (KEO, U-BJ, EL, and JL). After discussion, all authors agreed that saturation had been reached.

Step 2: The transcribed text was divided into units of meaning, which were condensed and labeled with codes and discussed (KEO, U-BJ, EL, and JL). The analysis was based on a manifest interpretation of the text.

Step 3: The various codes were compared, and similarities and differences were identified. The codes were then sorted into five categories. Thereafter, the two main categories were determined by consensus among all authors.

Step 4: The analysis was based on a manifest interpretation of the text. An overarching theme was identified when all authors performed a latent interpretation of the content.

## Results

The qualitative analysis identified two main categories: *perceptions of digital technology and the digital questionnaire in diabetes management and care* and *perceptions of participation in diabetes care* (Table 2). The overarching theme that emerged from the focus group discussions was *patients’ and professionals’ involvement in diabetes care using digital tools*.

**Table 2:** Theme, main categories, and categories.

Theme	Main categories	Categories
Patients' and professionals' involvement in diabetes care using digital tools	Perceptions of digital technology and the digital questionnaire in diabetes management and care	Hope and concern Opportunities and obstacles Individual needs and supportive structure
	Perceptions of participation in diabetes care	Give and take Trust and communication: A cornerstone for relationships

### Perceptions of Digital Technology and the Digital Questionnaire in Diabetes Management and Care

#### Hope and Concern

In terms of their perceptions of the use of digital technology in connection with the digital Diabetes Questionnaire and diabetes management and care, participants expressed both hope and concern. Patient focus groups expressed that using digital technology provides more information to the health care professionals. Regarding hope, the health care professional focus groups expressed that this form of care (digital technology) and the questionnaire could increase the availability of information required for retaining patients in the care unit. In addition, the health care professionals said that, with digital technology and the digital questionnaire, factors such as accessibility, individualization, and closer contact with the patient could lead to broader perspectives in individual encounters in diabetes clinics. In the patient focus groups, some argued that digital technology and the digital questionnaire would simplify life by providing opportunities to compile and analyze information on different aspects of living with the disease.

It’s a lot of technology now. And so, I think we must do this, to keep up with the younger generation because they are there. That’s where they communicate. You get so much more out of it. You get so much more information with the digital, you get many angles and lots to work with. Thus, you see so much more.

Health care professional

Participants in both the health care professional and the patient focus groups emphasized that this form of care (digital technology) can never take the place of physical encounters but that it should be an important complement to these encounters.

However, a concern was raised that health care professionals lack sufficient skills in digital technology, and that it was these professionals perceived to be responsible for stimulating the patients’ interest in this form of care. The professionals noted that the patients they meet have varying attitudes and experiences regarding using digital technology. The patient focus groups confirmed that there is a great variety of perspectives and attitudes regarding using and seeing the benefits of digital technology. Participants in both the health care professional focus groups and the patient focus groups expressed concern that a digital questionnaire would be perceived as violating the patient’s privacy, and some described a feeling of “being unprotected.” In addition, concerns about data confidentiality were expressed.

I see the possibilities in the technology. I am a technology lover myself in many respects—not least in my job. But I am terrified that, if you face this, the politicians will soon see it, and “Here, we can take and reduce the staff; here, we invest in digital technology instead.”

Patient

#### Opportunities and Obstacles

Both health care professionals and patients discussed and reflected on what opportunities the questionnaire provides in terms of improving patient care. The participants expressed that the digital questionnaire would probably create opportunities for person-focused approaches, as well as supporting an in-depth dialogue. It was also seen as clarifying caregivers’ interest in learning about what it is like to live with diabetes and facilitating discussion (such as allowing patients to talk about their moods). The participants argued that the digital questionnaire provides a more complete, overall picture that highlights individual needs and provides opportunities for preparation and feedback. They talked about a “feeling curve” and a “technical curve.” The digital questionnaire was seen as able to inspire support for a learning climate, which may, in turn, result in a new way of working. However, the participants also described obstacles such as the possibility that not all patients would appreciate the questionnaire and that there might be a lack of competency to follow up on the questionnaire or a lack of technical support provided by the health care professionals.

The patients expressed a lack of competency in diabetes care as potential obstacles. The professionals also expressed a lack of time as a potential obstacle. Nevertheless, the questionnaire was simultaneously seen as presenting a positive challenge because it could lead to reflection for both professionals and patients. For the professionals, it could mean a new approach and working method (improvement work or quality development). For the patients, the questionnaire could be an opportunity to talk and reflect on “What is important for me in my life with diabetes?”

Both patients and health care professionals stated that the questionnaire provides a good basis for the opportunity for monitoring and evaluation at the group level, which can create a foundation for positive changes in health care. In addition, the questionnaire provides a basis for comparing the results between health care units at the national level.

Nevertheless, I believe that it gives the patient the opportunity to reflect, when filling this in. … Therefore, that is a message we send with our questions actually…. And then I think it helps us to be able to sometimes, instead of talking blood sugar curves, get a little insight into what is behind this disease.

Health care professional

If we take this data in, we can’t just drop it there. We must find some structure for it too. And how to get this information, which can be a little worrying.

Health care professional

#### Individual Needs and a Supportive Structure

Both patients and health care professionals’ discussions identified two supportive structures as prerequisites for implementing the digital Diabetes Questionnaire. The first structure is the professionals’ need for an introduction and training before the start of the implementation. The second supportive structure is that the organization (eg, hospital, primary care clinic) provide infrastructure and other complementary resources to support the work and development of the diabetes care teams.

Education and training were not only requested at the beginning of the implementation but also as a continuous intervention. The health care professionals called for a clearer structure in their work to create well-functioning routines. The importance of support from the head of the department was emphasized to ensure that time and resources would be made available.

Getting enough time for the invitation for the diabetes care meeting, interpreting the answers, preparing for the meeting and for the actual meeting itself. Time is needed to structure the work before and create routines. And of course, support from the management.

Health care professional

### Perceptions of Participation in Diabetes Care

#### Give and Take

Primarily the health care professionals expressed the idea that patient participation in diabetes care is a requirement for change. They suggested that patient participation means that the health care professionals give support and make space for the patients to take responsibility for their own disease.

The patient focus groups also expressed that participation in their own diabetes care is a prerequisite for diabetes management and that the patient should be considered the principal actor in this process of providing the information about living with diabetes. Furthermore, the patients expressed the importance of having the right to decide for themselves and to be involved in the health care decisions based on their individual needs, which require both self-confidence and courage. They argued that there are different types of involvement, ie, participation in diabetes care, participation in diabetes care together with the health care professionals, and participation and involvement in the diabetes disease itself.

Furthermore, the professionals expressed the importance of the digital questionnaire in communicating the health care professionals interest in the patients’ responses to the questionnaire. The questionnaire signals that the diabetes health care professionals are interested in the patients’ life with diabetes. The patients expressed a similar idea in terms of participation being facilitated by the structure of the care meeting and that the questionnaire potentially increasing participation through structure and planning.

It must be those [patients] who control what actions they take. We are all consultants and informers, but participation is after all the Alpha and Omega.

Health care professional

It is a prerequisite for something to happen at all, that they are involved in it. Otherwise it doesn’t happen that much.

Health care professional

Used correctly, the questionnaire can probably increase participation. If it is, then there are enough questions about what support I need, and I express it. And that it is taken care of when resources are given to it; then it will increase my… or the care of my illness. This can’t be just a box-ticking affair, because that won’t increase the participation in care. Without it being like, you have a plan for how to proceed in these questions. And that it will be a continuation.

Patient

#### Trust and Communication: A Cornerstone for Relationships

The health care professionals’ focus groups expressed that trust and interaction between patients and health care professionals are the foundation for participation and a prerequisite for change. The patients expressed continuity as one cornerstone to build trust in their relationships with health care professionals. Another cornerstone for building these relationships is the interactive communication between patients and health care professionals. A prerequisite for interactive communication is speaking the same language and using the same vocabulary:

No, I think the patients may feel that they are being seen differently. That judgment ... than just coming to the visit, if they have now filled in for example this in at home, for example, before, and you bring it together, then maybe it becomes even clearer that you start from their needs, than when you just meet, I don’t know.

Health care professional

After all, it is a difference if you are to participate in an operation that you do once in your life, or if you are to participate in an illness that you will have for the rest of your life. I see it in a different way really, it is that you are the main person responsible for yourself and you should seek participation from others as support.

Patient

## Discussion

### Principal Findings

The main finding from the focus group interviews was the theme of patients and health care professionals’ involvement in diabetes care using digital tools. The main category of perceptions of digital technology and the digital questionnaire in diabetes management was built on the categories of hope and concern, opportunities and obstacles, and individual needs and supportive structure. The other main category, perceptions of participation in diabetes care, was built on categories of give and take, and trust and communication: a cornerstone for relationships. Although, the participants were not aware of the content of the Diabetes Questionnaire, they nevertheless expressed many views, thoughts, and feelings during the focus group interviews.

During the analysis of the focus group interviews, it became clear that the patients and health care professionals expressed similar expectations and reflections regarding both digital technology and the Diabetes Questionnaire. In particular, these similarities were seen to be prominent when the participants discussed digital technology in health care. Concerns such as a lack of knowledge about digital technology and a lack of privacy were evident. A previous study [21] has shown that the acceptance of digital technology relies on understanding of patients’ fears and concerns about lack of security. Lupton [22] highlighted the importance of reducing fears or concerns regarding insecurity among both patients and health care professionals through implementing secure computer systems and protecting personal data. Consequently, patients and health care professionals must both be aware of the security systems that surround digital technology in health care. Additionally, if these security systems are presented in a transparent and pedagogical way, knowledge of digital technology will increase and these concerns will decrease. Optimistically, in our study, the participants expressed hope that using digital technology can contribute to facilitating and supporting the everyday lives of people with diabetes. This finding is in line with previous studies [23-25] indicating that digital technologies can enhance diabetes care in a real-world setting.

When we analyzed the participants’ perceptions of the digital Diabetes Questionnaire, we found both differences and similarities between patients and health care professionals. The health care professionals saw an opportunity in that the Diabetes Questionnaire could facilitate person-centered care. They used phrases such as “an informed patient,” the opportunity for “person-centered work,” and “honest answers” from patients living with diabetes. The patients saw an opportunity for using the digital Diabetes Questionnaire to talk about and reflect on important factors in their everyday lives that influence their ability to take care of their diabetes. These results were in line with a newly published review showing that patient-reported outcome measures can facilitate a meaningful and focused conversation during the clinical encounter [8]. Conversely, patients in our study raised a concern about a lack of competency among health care professionals in terms of dealing with patients’ responses to the questionnaire. Furthermore, the participants emphasized the importance of a supportive structure. This highlights the importance of health care professionals receiving training on how to use the digital Diabetes Questionnaire and on how to sufficiently address patients’ responses to the questionnaire [26].

Furthermore, Greenhalgh et al [27] pointed out that the ways in which clinicians use patient-reported outcome measures are shaped by their relationships with patients. We found similar results under the category of trust and communication: a cornerstone for relationships. In addition, the participants reported that interactive communication between patients and professionals is important for building relationships. A prerequisite for interactive communication is speaking the same language and using the same vocabulary. There are recommendations for language used by health care professionals and others when discussing diabetes through spoken or written words [28]. An obstacle noted in our study, as well as in others, is that health care professionals lack time in everyday clinical care [29]. Both participant groups expressed that this lack of time could negatively affect the use of the digital Diabetes Questionnaire.

Perceptions of participation were discussed in the focus groups. The participants emphasized the importance of give and take in each care meeting for achieving participation. In addition, they reflected on whether participation meant that the patients become involved in their care or whether the health care professional must become involved in the patients’ daily life. The participants talked about adding the “feeling curve” from the digital Diabetes Questionnaire to the already existing “technological curve” to present a more holistic picture of the patients’ daily lives. The importance of participation and shared decision making in diabetes care and its association with optimal self-management has been shown in an earlier study [30]. Nevertheless, the participants in our study asserted that participation in diabetes care is suboptimal and perceived as an unclear concept.

Our focus group interview analysis showed that the participants expressed a positive attitude toward using the digital Diabetes Questionnaire. Discussing and reflecting on the questionnaire responses during physical encounters can improve diabetes care based on the person’s experience of living with diabetes and facilitate adequate support and self-management in a structured way. In addition, it is important to confirm the patient in a positive way. The response of the questionnaire clarifies what resources the patients have that could enable optimal daily diabetes self-management.

The analysis identified important factors to consider when introducing the digital Diabetes Questionnaire in clinical use. Both professionals and patients need support and training in the practical implementation of this intervention, and they should have an opportunity to provide feedback on the questionnaire answers. Öberg et al [31] pointed out that targeted training could increase the digital skills used in diabetes care. These needs are also confirmed by the participants’ discussion of the factor of insufficient time and the concern that patients’ responses to the questionnaire should be treated with great integrity. A review [14] in palliative care settings pointed out that providing an educational component prior to the implementation is crucial, in addition to interviewing health care professionals and patients.

### Limitations

Our sample may consist of participants with an interest in digital technology and in the development of diabetes care, which may have introduced bias. Thus, a sample using patients with low health literacy may show different results. Poor health literacy can seriously impair people’s interactions with health care professionals and their potential to benefit from digital health services. Qualitative studies are difficult to generalize because of small sample sizes. However, the results, especially on the perceptions of digital technology, are important for other groups of patients and for health care professionals in other disciplines.

### Implications

This study identified important factors to consider when introducing the digital Diabetes Questionnaire in clinical use and will serve as a basis for continued work in a larger implementation study for the Diabetes Questionnaire. When introducing digital patient-reported outcome measures such as the digital Diabetes Questionnaire, it is important to consider what conditions exist. A prerequisite is ensuring that both patients and health care staff members can handle digital technology. A second prerequisite is the careful evaluation of the content of the intended patient-reported outcome measure questions. Finally, a trusting relationship between health care professionals and patients is required to make conversations based on the digital patient-reported outcome measure feel meaningful.

## Appendix

Multimedia Appendix 1 Focus group interview guide.
