# Supplementary material for: Patients’ and Health Care Professionals’ Perceptions of the Potential of Using the Digital Diabetes Questionnaire to Prepare for Diabetes Care Meetings: Qualitative Focus Group Interview Study
Source: J Med Internet Res. 2020 Aug 19;22(8):e17504. doi: 10.2196/17504 (PMC7468633; doi:10.2196/17504)
Supplement: Multimedia Appendix 1 [file jmir_v22i8e17504_app1.docx]

Appendix 1.

**Focus group interview guide for the health care professionals and the patients**

**Group interview guide for the health care professionals before the implementation of the Diabetes Questionnaire**

Experience with the National Diabetes Register (NDR) and thoughts regarding the introduction of the digital Diabetes Questionnaire on how the patient is feeling, how the patient is managing diabetes, and is experiencing support from the health care system.

- How do you use the NDR? *Probes:* *Can you elaborate on your answer? Can you give an example?*
- How confident do you feel about using the NDR?
- What are the advantages and disadvantages of using the NDR?
- What do you think about using digital technology in diabetes care?
- What do you know about using a digital questionnaire in the diabetes care clinic visits?

What are the attitudes regarding the digital tool (Diabetes Questionnaire) and the use of the patient-reported information about how the patient is feeling, managing diabetes and experiencing support from the health care system?

- How do you feel about the introduction of a digital questionnaire in diabetes care?
- How do you think the patient visit will be affected by using the questionnaire?
- What are the advantages and disadvantages of using the questionnaire?
- How do you think diabetes care can be developed with the help of the questionnaire responses?
- How do you think about the concept of participation in diabetes care?
- How can the questionnaire affect the patient's participation and control over their health and care?
- What support do you think you need when introducing the digital questionnaire in order to provide good care?
- What resources do you think you need when introducing the digital questionnaire?

Final questions

- Finally, is there anything else you would like to add?
- What was the most important thing we have talked about today?

**Focus group interview guide for the patients before the implementation of the Diabetes Questionnaire**

Experience with the National Diabetes Register and thoughts regarding the introduction of the digital Diabetes Questionnaire

- What do you know about the NDR?
- If you use the NDR, how do you use it?
- What are the advantages and disadvantages of the NDR?
- What do you think about using digital technology in diabetes care?
- What do you know about using a digital questionnaire that measures how you feel, manages your diabetes and experiences support from the health care system, during a visit to the diabetes clinic?

What are the attitudes regarding the digital tool (Diabetes Questionnaire) and the use of patient-reported information about how you, as a person with diabetes, feel, manage your diabetes, and experience the support from the health care system?

- How do you feel about the introduction of a digital questionnaire in diabetes care?
- How do you think the visit will be affected by using the questionnaire?
- What are the advantages and disadvantages of using the questionnaire? The information in the questionnaire (how you feel, manage your diabetes and experience the support from the health care system)? The digital questionnaire itself?
- How do you think diabetes care can be developed with the help of the questionnaire responses?
- How do you think about the concept of participation in diabetes care?
- How can the s questionnaire affect your participation and control over your health and care?

Final questions

- Is there anything else you would like to add?
- What was the most important thing we talked about today?
